# Cell wall as a target for bacteria inactivation by pulsed electric fields

**DOI:** 10.1038/srep19778

**Published:** 2016-02-01

**Authors:** Flavien Pillet, Cécile Formosa-Dague, Houda Baaziz, Etienne Dague, Marie-Pierre Rols

**Affiliations:** 1 CNRS; IPBS (Institut de Pharmacologie et de Biologie Structurale); 205 route de Narbonne BP64182, F-31077 Toulouse, France; 2 Université de Toulouse; UPS; IPBS; F-31077 Toulouse, France; 3 CNRS, LAAS, 7 avenue du Colonel Roche, F-31400 Toulouse, France; 4 CNRS, UMR 7565, SRSMC, Vandœuvre-lès-Nancy, France; 5 Université de Lorraine, UMR 7565, Faculté de Pharmacie, Nancy, France

## Abstract

The integrity and morphology of bacteria is sustained by the cell wall, the target of the main microbial inactivation processes. One promising approach to inactivation is based on the use of pulsed electric fields (PEF). The current dogma is that irreversible cell membrane electro-permeabilisation causes the death of the bacteria. However, the actual effect on the cell-wall architecture has been poorly explored. Here we combine atomic force microscopy and electron microscopy to study the cell-wall organization of living *Bacillus pumilus* bacteria at the nanoscale. For vegetative bacteria, exposure to PEF led to structural disorganization correlated with morphological and mechanical alterations of the cell wall. For spores, PEF exposure led to the partial destruction of coat protein nanostructures, associated with internal alterations of cortex and core. Our findings reveal for the first time that the cell wall and coat architecture are directly involved in the electro-eradication of bacteria.

The bacterial cell wall is the main barrier against the environment^1^ and also confers the bacteria their shape and prevents cell rupture and osmotic shock^2^. Peptidoglycan is the major constituent of the cell wall and its architecture in the cell-wall network contributes to bacterial resistance^3^. The cell wall is therefore the main target for bacterial inactivation with numerous antibiotics having been developed with this aim^4^. Antimicrobial peptides can be used to form pores and to inhibit cell-wall synthesis leading to microbial killing 5,6. Another strategy currently used in food disinfection^7^,^8^ consists of Pulsed Electric Field (PEF) treatment. However, PEF is considered to be inefficient for the eradication of bacterial spores^9^. This limit prevents PEF from being widely adopted as an efficient and safe means for food and water sterilization. Currently, PEF is not used in hospitals to treat bacterial infection, mainly because of a lack of understanding of how the inactivation process actually works. Fundamental research on PEF has, nevertheless, led to highly efficient cancer therapies^10^,^11^.

Here, we first define the PEF parameters required to kill both vegetative bacteria and spores. Then, we report the inactivation process. Current dogma considers the cell membrane as the unique target of bacterial inactivation during Pulsed Electric Field (PEF) exposure^12^. Our studies have cast doubt on this. However, the bacteria’s main barrier against the environment, i.e. the cell wall for vegetative bacteria and the coat for spores, is not covered in the standard description of the PEF inactivation process.

To fill this gap, we used multiparametric Atomic Force Microscopy (AFM)^13^ a technique which allows the nanoscale observation of live single cells. AFM provided, height maps^14^, stiffness maps^15^ and hydrophobicity maps by Chemical Force Microscopy^16^,^17^. Here, we performed nanoscale investigations of the damage induced by PEF-exposure in *Bacillus pumilus*, a non-pathogenic model of food contaminants like *Clostridium difficile*, *Clostridium botulinum, Bacillus cereus*^18^,^19^. For vegetative bacteria, the peptidoglycan network is organized in cables perpendicular to the long axis of the cell^3^. The presence of similar structures visualized in AFM stiffness maps suggested the presence of peptidoglycan cables and highlighted the importance in the maintenance of global cell-wall rigidity. After PEF, this cable network became disorganized. Furthermore, variation of electric potential and hydrophobicity suggested general molecular disorganization of the cell wall. Scanning electron microscopy (SEM) confirmed the morphological damage to the cell wall. Furthermore, transmission electron microscopy (TEM) revealed degradation of the entire cell envelope with the impossibility to distinguish the plasma membrane from the cell wall. Bacterial death results in leakage of nucleic acids^20^ and osmotic shock due to cell-wall disorganization. For spores, Plomp previously reported observing ridges using AFM in air^21^, which he attributed to an artefact due to drying. Here, we revealed the presence of similar ridges by AFM in liquid (no drying step). These structures are therefore not drying artefacts and probably play a role in functional adhesion of spores to surfaces. In addition, TEM demonstrated that PEF cleaves the protein layers of the coat destroying the ridges.

## Results

### Correlation between electro-permeabilisation and bacterial inactivation

Bacteria were pulsed in water with a conductivity of 500 μS/cm, similar to domestic water. Inactivation was calculated by colony counting. Reversible and irreversible permeabilisation rates were evaluated by propidium iodide (PI) staining^22^ as shown in Supplementary Fig. S1.

**Figure 1:**
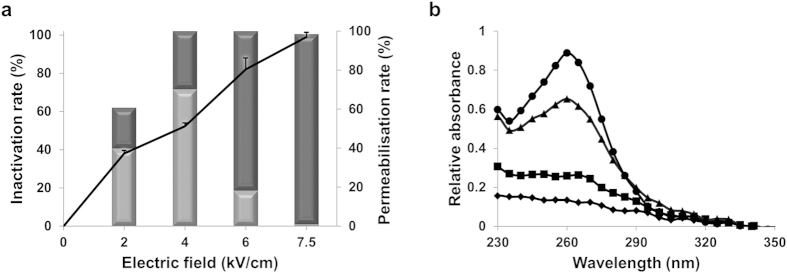
Cell membrane permeabilisation induced inactivation of vegetative bacteria. **(a)** Effect of 1000 pulses at different electric field strengths on inactivation of the vegetative form (black curve) and the percentage of plasma membrane permeabilisations (light diagram for reversible and dark diagram for irreversible). **(b)** Nucleic acids released according to electric field wavelength length applied. Absorbance spectra of the supernatant solution of vegetative bacteria having received 1000 pulses at 2kV/cm, diamonds; 4 kV/cm, squares; 6 kV/cm, triangles; 7.5 kV/cm, circles. The absorbance values were obtained after subtraction of supernatant values of untreated bacteria. All experiments were performed in triplicate.

We first explored the influence of the electric field strength on the inactivation of vegetative bacteria by applying 1000 pulses of 5 μs from 2 to 7.5 kV/cm (Fig. 1a). Inactivation varied from 38 ± 1% to 98 ± 2%. Between 2 and 6 kV/cm, inactivation was partially due to reversible permeabilisation. In contrast, for the strongest electric field used (7.5 kV/cm), inactivation was entirely due to irreversible permeabilisation. This was correlated to concomitant release of nucleic acids as shown in Fig. 1b.

For bacteria under spore form, 1000 pulses at 7.5 kV/cm were insufficient to achieve inactivation. Inactivation increased with the number of pulses, reaching 67 ± 8% at 10 000 pulses (Supplementary Fig. S2).

### Cell wall deterioration in vegetative bacteria

In order to address the structure/function relationship, we implemented various microscopy techniques to characterize nanoscale cell wall damage. SEM was used to inspect the cell surface. TEM showed the interior of the bacteria. Multiparametric AFM on living cells was carried out to correlate nanoscale observations with nanomechanical and physical properties.

**Figure 2:**
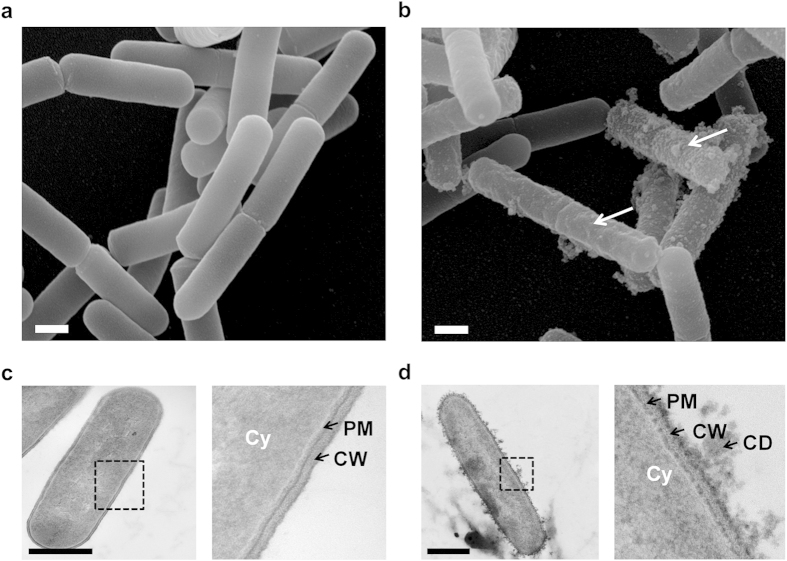
PEF-induced cell wall disruption on vegetative bacteria. **(a)** SEM image showing population of untreated bacteria. Bacteria are smooth. **(b)** Pulsed bacteria were visualized by SEM after PEF exposure (1000 micropulses at 7.5 kV/cm). White arrows indicate surface damage. **(c)** TEM image of untreated *Bacillus pumilus*. Inset shows bacterial architecture with the cytoplasm (Cy), plasma membrane (PM) and cell wall (CW). **(d)** TEM image of a pulsed bacterium after PEF. Inset shows the expulsion of cell-debris (CD), damage to the PM and the CW. Scale bars: 500 nm.

We first visualised the morphology of entire vegetative bacteria. Control cells, imaged by SEM, revealed smooth surfaces (Fig. 2a). TEM images displayed the organization expected for gram-positive bacteria with a clearly defined cytoplasm (Cy), plasma membrane (PM) and cell wall (CW)(Fig. 2c). After PEF exposure, heterogeneous populations of cells were detected, some presenting extensive surface damage (Fig. 2b). TEM images revealed the presence of cell-debris and significant damage to the PM and CW (Fig. 2d).

**Figure 3:**
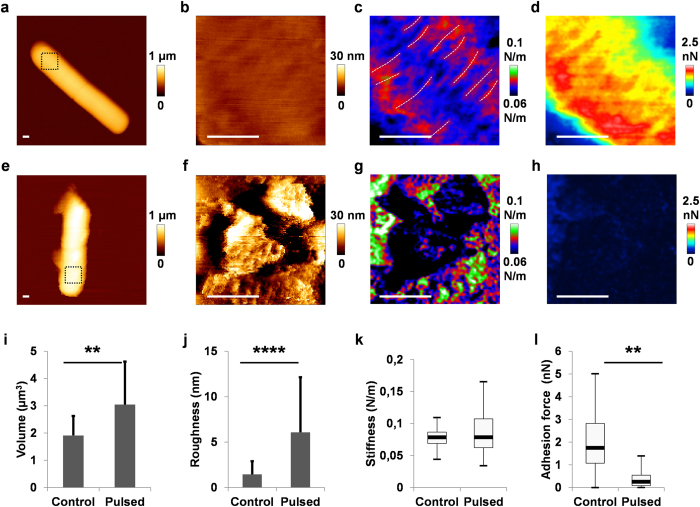
PEF-induced morphological, mechanical and physical damage to cell wall. **(a,e)** Examples of vegetative bacteria visualized by AFM; inset shows location of height resolution image **(b,f)**, stiffness map **(c,g)** and adhesion force map **(d,h)** measured by hydrophobic interactions; from an untreated bacterium **(a–d)** and a pulsed bacterium **(e–h)** with 1000 micropulses at 7.5 kV/cm. Scale bars: 200 nm. **(i)** Statistical analysis of the bacterial volume. PEF exposure induces swelling of bacteria. The measurements for each condition were performed on four replicates with at least 30 bacteria each. **(j)** Statistical analysis of roughness. Roughness increased after PEF. The roughness values (Ra) were calculated for each condition from 30 bacteria in four experiments. **(k)** Statistical analysis of the stiffness. PEF increased the heterogeneity of stiffness. Box plots were obtained from stiffness maps of 1024 curves from 15 bacteria per condition. **(l)** Statistical analysis of hydrophobicity. Loss of hydrophobicity was noted after PEF. For each condition, box plots were determined with adhesion maps of 16384 curves from seven bacteria from two independent experiments.

These observations were supported by AFM in liquid. Indeed, height images showed homogeneous bacteria in controls (Fig. 3a,b) and a strong heterogeneity after PEF exposure (Fig. 3e,f). Morphological properties were evaluated by statistical analysis for each condition from 30 height AFM images. The mean volume was 1.9 ± 0.7 μm^3^ for control bacteria and 3.0 ± 1.6 μm^3^ for PEF-treated bacteria (Fig. 3i). This 60% increase in volume suggested osmotic shock probably due to cell wall disruption. At the nanoscale, a mean roughness of 1.4 ± 0.5 nm was determined for untreated bacteria and 6.1 ± 5.2 nm after PEF exposure (Fig. 3j). This Increased roughness suggested disorganization of the cell wall surface and correlates with the cell debris observed by SEM and TEM.

We next investigated the mechanical and hydrophobic properties of the cell walls. To study the mechanical properties, successive force measurements applied 1 nN with the AFM tip. We quantified the mechanical properties of the bacteria via the slope of the force-distance curves (see Materials and Methods), henceforward referred to a stiffness”. For untreated bacteria, the stiffness map revealed cables running around the cylindrical cell-wall (Fig. 3c; dotted lines), as recently described with the cables of peptidoglycan by Vollmer and Seligman^3^. After PEF, the cables were absent from the stiffness images (Fig. 3g). AFM revealed a local decrease of stiffness on cell debris and an increase of heterogeneity. To justify statistical analysis, we determined stiffness on 15 different untreated bacteria in 3 independent experiments (Fig. 3k). For untreated bacteria, the homogeneity was confirmed by the Gaussian distribution of values exhibited by the box plot, with a mean of 0.08 ± 0.01 N/m. In contrast, PEF treatment of vegetative bacteria was correlated with an increase in heterogeneity (0.09 ± 0.03 N/m). In addition, the surface hydrophobicity of the cell walls was evaluated by chemical force measurements with the AFM tip functionalized with −CH_3_ groups^16^,^17^. The adhesion maps (16 384 force curves) revealed greater hydrophobicity in untreated bacteria (Fig. 3d), disappearing after PEF exposure (Fig. 3h). Analysis of seven independent bacteria (114 688 adhesion curves) confirmed a statistical difference (Fig. 3l), with an adhesion force of 2.1 ± 1.2 nN for untreated bacteria which is similar to adhesion forces measured on microorganisms with 25% of CH3 surface fraction^17^ and 0.2 ± 0.2 nN for pulsed bacteria. In addition, electrophoretic mobility measurements revealed an increase in the surface charge (zeta potential increased from −34 mV in controls to −28 mV after PEF (Supplementary Fig. S3). These results revealed morphological, mechanical and physical damage to the cell wall.

### Spore coat deterioration

**Figure 4:**
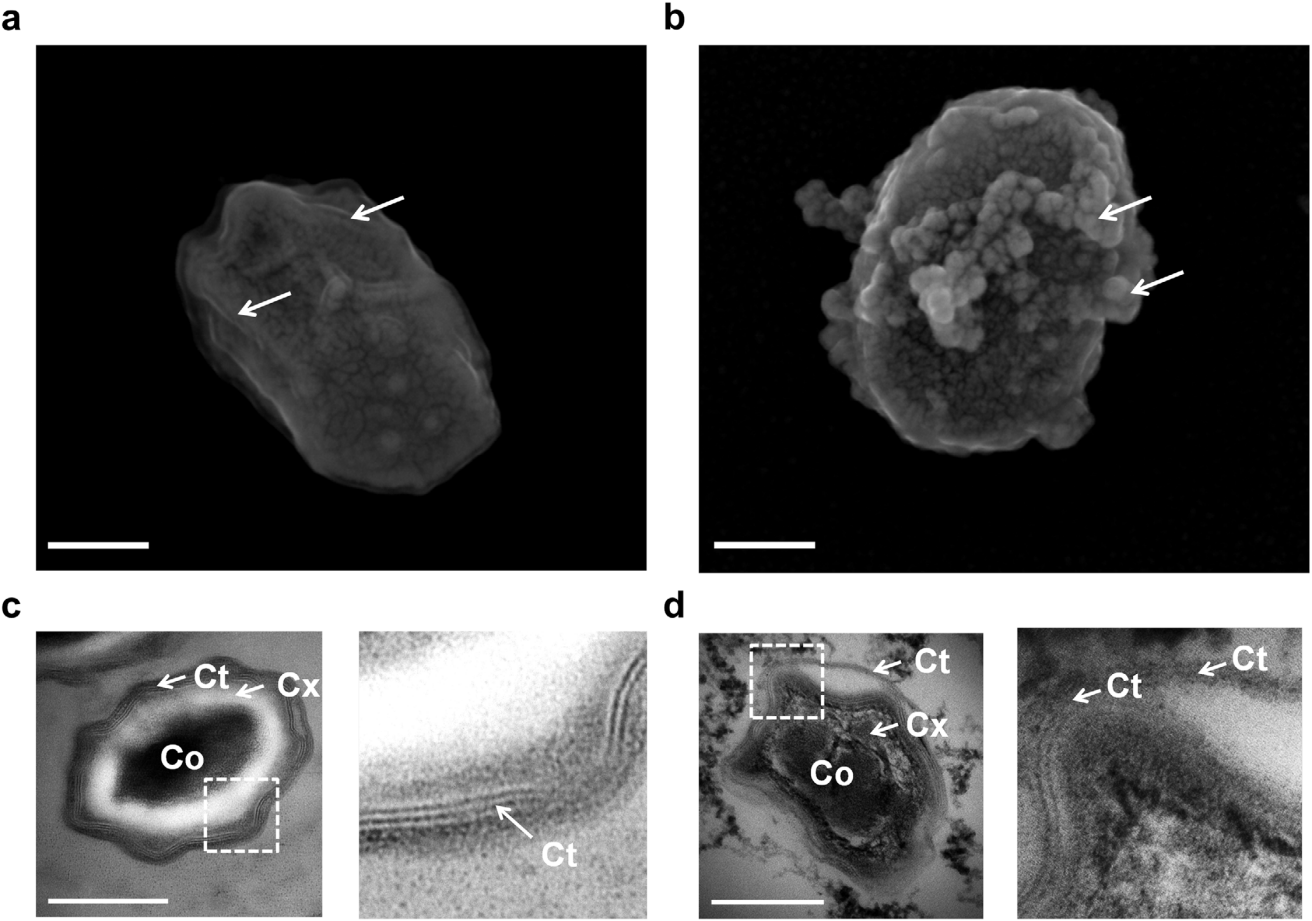
PEF induced internal damage to spores. **(a)** SEM image of a typical untreated spore. Ridges are indicated by the white arrows. **(b)** Example of a pulsed spore visualized by SEM after 10 000 micropulses at 7.5 kV/cm. Budding structures were observed. **(c)** TEM images of an untreated spore. The core (Co), the cortex (Cx) and the coat (Ct) are observable. Inset display nanostructures of internal coat organized in protein multilayers. **(d)** TEM image of a pulsed spore. Internal damage is seen in core and cortex. Inset indicates the cleavage of protein multilayers in the coat. Scale bars: 250 nm.

Spores were analysed by SEM and TEM (Fig. 4). Parallel nanostructures were discernible by SEM on the coat of untreated spores (Fig. 4a). These protein nanostructures named ridges are classically described on bacillus spore coats after desiccation^23^,^24^. After PEF, budding structures were detected by SEM (Fig. 4b). TEM images displayed the ultrastructure of the spores (Fig. 4c). The core (Co), the cortex (Cx) and the coat (Ct) are perfectly distinguished, as described by Tokuyasu and Yamada in *B. subtillis*^25^. The insets indicate the coat architecture, arranged in protein multilayers^26^. These structures are folded in ridges and previously described, in air, on *Bacillus* spores^23^,^27^. After PEF exposure, the ultrastructure of the spores was disturbed (Fig. 4d). Internal damage was observed inside the core and the cortex compartments. In addition, the inset shows cleavage of the coat protein multilayers.

**Figure 5:**
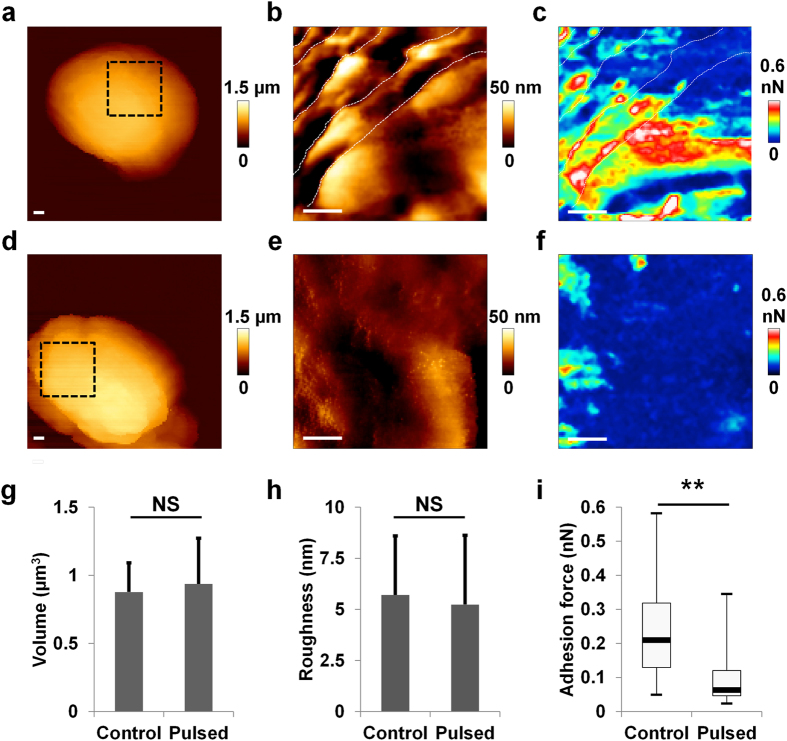
PEF decreased the hydrophobicity of spores. **(a,d)** Example of AFM image of spores; inset shows localization of height resolution image **(b,e)** and adhesion force map **(c,f)**; from an untreated spore **(a–c)** and pulsed spore **(d–f)** with 10 000 micropulses at 7.5 kV/cm. Scale bars: 100 nm. **(g)** Statistical analysis of spore volume. There is no variation after PEF exposure. The measurements were performed on four replicates per condition on at least 25 spores. **(h)** Statistical analysis of roughness. The roughness is similar between untreated and pulsed spores. The diagram was obtained with 25 spores per condition, from four independent experiments. **(i)** Statistical analysis of hydrophobicity. A decrease of hydrophobicity was measured after PEF. For each condition, box plots were drawn from adhesion maps of 16 384 curves from seven spores in two independent experiments.

The presence of ridges on the coat was confirmed by AFM in liquid (Fig. 5a,b; Supplementary Fig. S4). After PEF, the ridged structures were absent and damage visible on the spore coat (Fig. 5d,e). Roughness and volume were calculated from 25 AFM images. The volume was 0.9 ± 0.2 μm^3^ for untreated and 0.9 ± 0.3 μm^3^ for PEF-exposed spores (Fig. 5g). Mean roughness (R_a_) was 5.5 ± 2.9 nm for untreated spores and 4.2 ± 1.7 nm after PEF exposure (Fig. 5h). The absence of swelling was probably due to the dehydrated nature of the spores.

On investigating the mechanical and physical properties, it was found that the stiffness of the spores was over 15 times greater than that of vegetative bacteria without any difference after PEF exposure (Supplementary Fig. S5). Hydrophobicity was evaluated by chemical force measurements, as described earlier. The adhesion maps exhibited a decrease in hydrophobicity between untreated spores (Fig. 5c) and pulsed spores (Fig. 5f). Significant differences were confirmed by statistical analysis (Fig. 5i). In addition, a huge inversion of zeta potential was measured, from −47 mV in controls to +20 mV after pulses (Supplementary Fig. S3).

## Discussion

Our experiments using advanced microscopy reveal the influence of the cell wall for vegetative bacteria and the coat for spores, on the viability of *B. pumilus* after PEF exposure.

The cell wall is a vital component of vegetative bacteria, responsible for cell shape^28^ and for maintaining the intracellular contents and turgor pressure inside the cell^29^. Morphological imaging by SEM revealed alterations of cell shape and the presence of cell debris after PEF exposure. AFM in liquid confirmed these observations, with an increase of bacteria volume. The swelling suggests wall degradation with rupture of the turgor pressure, inducing osmotic shock. TEM images confirmed this: drastic modifications were noted inside the cell envelope with the impossibility of clearly distinguishing the cell wall and the plasma membrane. Consequently, the cell wall became permeable and the presence of nucleic acids in the extra cellular medium confirmed cytoplasm leakage. Different types of degradation can explain cell wall permeability after PEF exposure. Firstly, high-resolution AFM revealed loss of the stiff cables and a decrease of cell wall rigidity. These results suggested a destabilization of the peptidoglycan network. This architecture is essential for gram positive bacteria and constitutes the first barrier against a hostile environment^30^. Secondly, the loss of hydrophobicity as determined by chemical force measurement is concomitant with the entrance of hydrophilic molecules, like propidium iodide. These observations suggest degradation of molecular compounds, present at the cell-wall surface of *Bacillus* species. Thirdly, a variation of the zeta potential was noted. On the cell wall of gram positive bacteria, the phosphoryl groups of teicoic acids are responsible of the surface charge^31^. It is therefore appropriate that degradation of the cell wall led to a variation of the zeta potential.

In spores, the cell envelope, arranged in successive multilayers, confers high resistance to PEF exposure^9^,^32^. SEM images revealed the presence of ridged structures on the coat surface. They were previously described in the literature as being artefacts of spore desiccation^21^. This statement is in contradiction with our observations. Here, the presence of ridges was visualized by AFM in liquid. These structures could be involved in invasive properties via an increase of adhesion onto solid surfaces. After PEF exposure, SEM images revealed the presence of cell debris on the coat surface. TEM confirmed this and extensive internal damage was visualized on the core, cortex and coat. However, unlike in vegetative bacteria, neither DNA release nor swelling was detected. The dehydrated nature of spores and highly condensed DNA limit exchanges between core and extracellular medium. At the nanoscale, topographic imaging by AFM revealed damage on the coat surface after PEF exposure, principally on the protein ridges. The damage suggested a direct or an indirect effect of PEF on the protein architecture of the coat. We evaluated this hypothesis by coat surface measurements of hydrophobicity and electric potential. These characteristics are primordial for spore adhesion and confer their invasive properties^33^. Chemical force measurements were therefore performed by AFM. Interestingly, the hydrophobicity was lower than that of vegetative bacteria. This feature is due to the absence of exosporium in the *B. pumilus* spore^34^. However, a three-fold decrease of hydrophobicity was noted after PEF exposure. In addition, PEF led to a zeta potential inversion. These results suggest a drastic alteration of spore coat constituents such as the proteins spoIVA and spoVM which have a major effect on coat structure^26^.

Many questions remain unanswered. The chronology of events during PEF inactivation is still unknown. For vegetative bacteria, the degradation of the cell wall may be due to a direct consequence of PEF exposure and indirect disorganization due to cell membrane permeabilisation. For spores desiccation prevents cell-membrane permeabilisation. In this case, the spore coat (principally made of proteins) is a key factor. The degradation of proteins by PEF is poorly described in the literature and should be investigated.

In summary, we report here evidence that PEF exposure not only induces plasma membrane permeabilisation but also affects cell wall integrity for vegetative bacteria and leads to changes in coat, cortex and core for spores. This is the first time it has been shown as the main cause of loss of viability. We indeed observed molecular disorganization which allowed inactivation of bacteria. The drastic physical modifications of the surface decreased the invasive properties of the bacteria. These results open a new avenue for inactivation of bacteria by direct targeting of the cell wall.

## Materials and Methods

### Growth conditions, preparation of vegetative and spore bacteria

The *Bacillus pumilus* strain (ATCC 27142) was used for this study. Vegetative cells were cultivated at 37 °C in Luria broth (Sigma-Aldrich, France) and collected in exponential growth phase (1.8 ≤ OD_600_ ≤ 2). After centrifugation for 5 min at 6000g, the pellet was resuspended in 4.1mM of NaCl. Sporulation was carried out for 5 days at 37 °C in Difco Sporulation Medium (DSM), as described by Schaeffer *et al.*^35^. The remaining vegetative bacteria were inactivated by heating at 80 °C for 20 min and lysed in 50 μg/ml of lysozyme in 50 mM Tris-HCl, pH6.2, for 1h at 37 °C. Spores were collected by centrifugation (5 min, 10 000 g) and washed in deionized water, in 0.05% SDS and again in deionized water 3 times. The spores were kept in solution at 4 °C prior to experiments.

### Pulsed Electric Field

Pulses were generated by a GHT-U square wave generator (β-tech, France) and recorded on a Tektronix TDS5104B oscilloscope (Tektronix, USA). Electroporation cuvettes of 100 μl with an inter-electrode distance of 1 mm were used (Eppendorf, France). For all experiments, we applied 5 μs-pulses at a frequency of 1 kHz in a 4.1 mM NaCl solution, with a conductivity of 500μS/cm and a pH of 7. Two parameters were varied, the electric field (from 2 kV/cm to 7.5 kV/cm) and the number of pulses (from 1000 to 10 000). The output voltage from the square wave generator was closely followed for each experiment to know the electric field actually delivered to each cuvette. To prevent an increase of temperature, a delay of 1 min was applied between each series of 1000 pulses. A constant temperature was observed with a laser thermometer (data not shown). To limit the production of electrolytes, the polarity was manually inverted every 500 pulses.

### Evaluation of inactivation rate

Following PEF-exposure in liquid, bacteria were spread on LB agar petri dishes. Colonies were counted after incubation overnight at 37 °C. The proportion of inactivation was given as log10 of the ratio between untreated and PEF-treated bacteria. For each condition tested, 3 independent experiments were performed with a total of 9 petri dishes per analysis.

### Fluorescence microscopy

Bacteria were observed by fluorescence under a Leica DMIRB microscope with a X100 oil-immersion objective (Leica, Germany). The wavelengths were selected by using the ET-mCherry filter (540 nm ≤ λex ≤ 580 nm; dichromatic mirror pass band, 595 nm ≤ λem ≤ 640 nm). The permeabilised bacteria were visualized by staining with 100 μM propidium iodide (PI) (Supplementary Fig. S1). For each condition, measurements were made on around 500 bacteria in 3 independent experiments. The percentage of total permeabilisation was evaluated in the presence of PI just after the pulses (t0), irreversible permeabilisation was measured by addition of PI 30 min after the pulses (t30). The reversible permeabilisation was taken to be the difference between total permeabilisation and irreversible permeabilisation.

### Nucleic acid quantification

To measure nucleic acid release, following PEF exposure at different field strengths, vegetative bacteria were centrifuged at 6000g for 3 min. The supernatant was analysed for UV-absorption with a Nanodrop 1000 (*Thermo Scientific*). The wavelength range was from 230 nm to 350 nm. The curves were obtained after subtraction of UV-absorbance from the supernatant of untreated bacteria. The amount of nucleic acids released was evaluated at 260 nm.

### AFM experiments

Vegetative bacteria and spores were immobilized for 1 h on a glass slide coated with polyethylenimine, then washed and kept under 4.1 mM NaCl during the experiment. AFM measurements were performed with a Nanowizard III (JPK Instruments, Germany) in Quantitative Imaging mode (QI)^36^and Force Volume mode (FV). For QI, MLCT cantilevers (Bruker, Germany) were used with a spring constant measured at around 0.5 N/m. For FV mode, mechanical studies were performed with MLCT cantilevers for vegetative bacteria and PPP-NCH cantilevers (Nanosensors, Switzerland) for spores, with a spring constant of around 40 N/m. The forces applied to bacteria during FV measurements were 1 nN for vegetative cells and 10 nN for spores. An example of Force curves used for stiffness calculation is shown in Supplementary Fig. S6, according to the equation Kcell = K(S/1-S) where K is the cantilever spring constant and S the slope of the experimental force versus distance curve^37^. Roughness values (Ra) were calculated from square height images of 500 μm. Adhesion values were performed with NPG-10 gold cantilevers functionalized by 1-Dodecanethiol (Sigma-Aldrich, France), as the procedure described previously by Alsteens *et al.*^16^. All AFM analyses were performed with the JPK Data processing software (version 5.0.53).

### SEM and TEM observation

In order to observe bacterial damage by electron microscopy, bacteria were fixed with 2% glutaraldehyde in 0.1M Sorensen phosphate buffer pH7.2, just after PEF-exposure. The samples were prepared by the CMEAB platform (Toulouse, France). Images were visualized in SEM with an electron microscope Quanta^TM^ 250 FEG (FEI, USA) at an accelerating voltage of 15 kV. For TEM, ultrathin sections, 70 nm thick were placed on grids. Images were acquired with HT 7700 at 80 kV (Hitachi, USA).

### Statistical analysis

For more relevant statistical analysis, we performed Welch’s t-test which is similar to Student’s t test but more suitable for unequal variances.

## Additional Information

**How to cite this article**: Pillet, F. *et al.* Cell wall as a target for bacteria inactivation by pulsed electric fields. *Sci. Rep.* **6**, 19778; doi: 10.1038/srep19778 (2016).

## Supplementary Material

Supplementary Information
