# Supplementary material for: Cell wall as a target for bacteria inactivation by pulsed electric fields
Source: Sci Rep. 2016 Feb 1;6:19778. doi: 10.1038/srep19778 (PMC4735277; doi:10.1038/srep19778)
Supplement: Supplementary Information [file srep19778-s1.pdf]

Cell wall as a target for bacteria inactivation by pulsed electric fields

Flavien Pillet[1,2], Cécile Formosa-Dague[2,3,4,5], Houda Baaziz[1,2], Etienne Dague[1,3]*, Marie-Pierre Rols[1,2]*

[1] CNRS; IPBS (Institut de Pharmacologie et de Biologie Structurale); 205 route de Narbonne BP64182, F-31077 Toulouse, France

[2] Université de Toulouse; UPS; IPBS; F-31077 Toulouse, France

[3] CNRS, LAAS, 7 avenue du Colonel Roche, F-31400 Toulouse, France

[4] CNRS, UMR 7565, SRSMC, Vandœuvre-lès-Nancy, France

[5] Université de Lorraine, UMR 7565, Faculté de Pharmacie, Nancy, France

*Corresponding authors contributed equally to the work

Etienne Dague: edague@laas.fr

Marie-Pierre Rols : rols@ipbs.fr

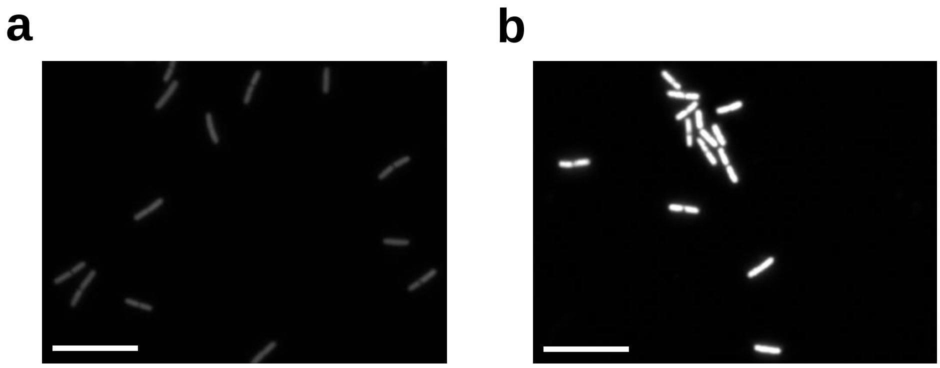

Supplementary Fig.S1. Fluorescent microscopy of vegetative *B.pumilus* in presence of 100 µM of propidium iodide. (a) Example of bacteria in control condition and **(b)** after 1000 micropulses at 7.5kV/cm. Scale bars: 10 µm.

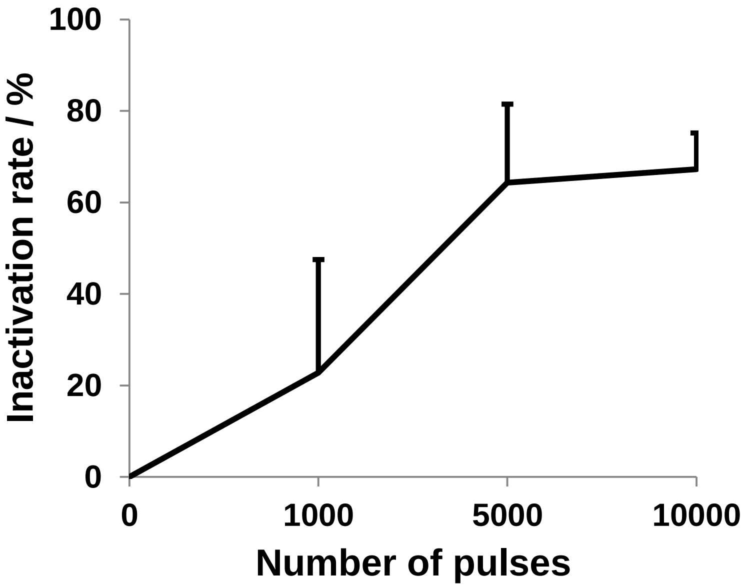

Supplementary Fig. S2. Determination of spore inactivation according to the number of pulses of 5µs at 7.5 kV/cm. The inactivation rate was evaluated by colony counting.

	Control	Pulsed	Statistic difference
Vegetative	-34 ± 0.3	- 28 ± 2.1	****
Spore	-47 ± 1.0	+20 ± 0.2	****

Supplementary Fig. S3. Zeta potential of control and pulsed bacteria. Each value(mV) were obtained from 9 independent mesurements from 3 independent bacteria cultures.

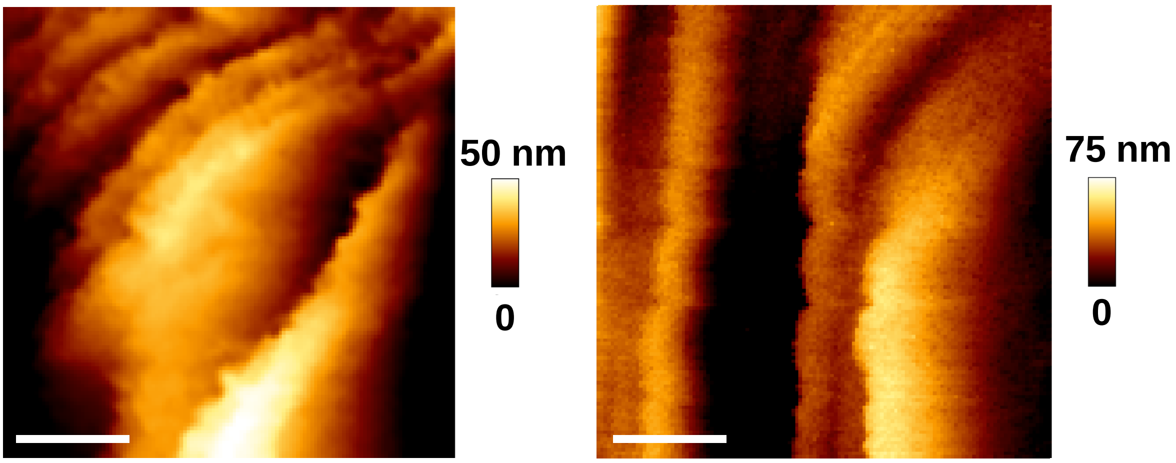

Supplementary Fig. S4. Examples of ridges on untreated spores observed by AFM in liquid. Scale bars: 100 nm.

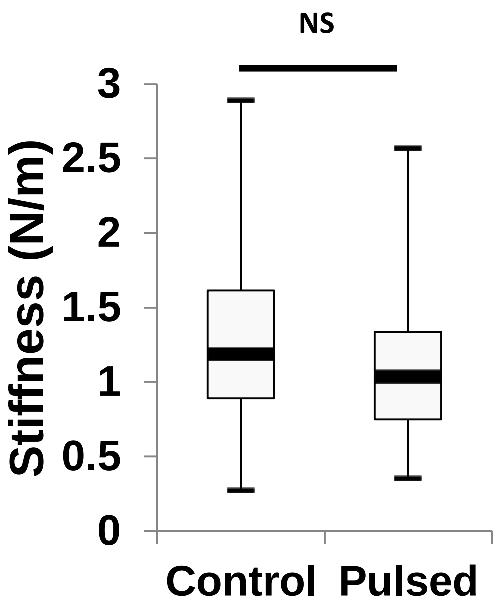

Supplementary Fig. S5. Statistical analysis of spore stiffness. Box revealed no significant differences of stiffness after PEF-exposure. The box plots were obtained with 15 spores from 3 independent experiments.

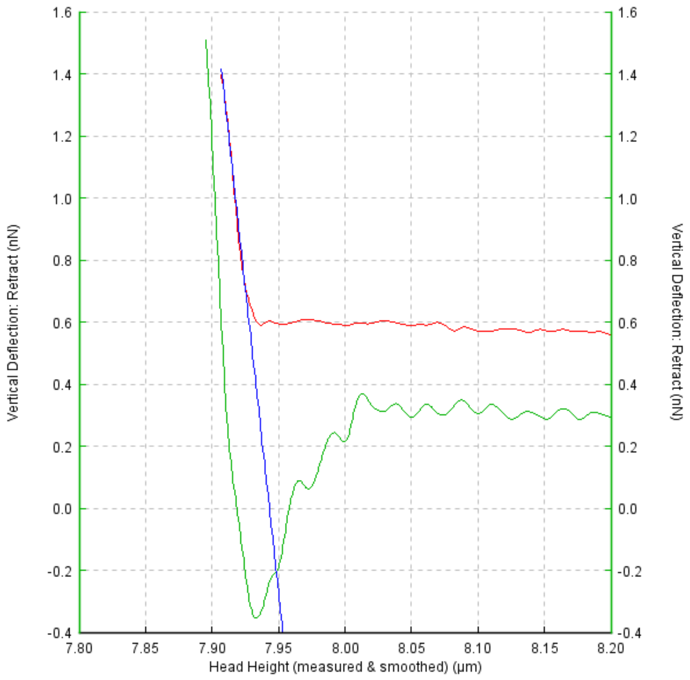

Supplementary Fig. S6. Example of Force distance curve used for stiffness calculation. The stiffness was calculated with the approach curve (red line) on 10 nm of indentation. The slope is shown in blue. The retract curve is exposed in green line.
